# Electroanalytical Overview: the Measurement of Diuron

**DOI:** 10.1021/acsmeasuresciau.5c00109

**Published:** 2025-09-18

**Authors:** Robert D. Crapnell, Craig E. Banks

**Affiliations:** Faculty of Science and Engineering, 5289 Manchester Metropolitan University, Dalton Building, Chester Street, Manchester M1 5GD, Great Britian

**Keywords:** diuron, electroanalysis, sensors, electrochemistry, 3D printing, molecular imprinted polymers

## Abstract

Diuron, a widely used herbicide, has been banned or heavily restricted in several countries due to its environmental persistence and toxicity to aquatic ecosystems. Its chemical stability allows it to remain in soil and water for extended periods, leading to long-term contamination and potential leaching into groundwater. This is particularly concerning because diuron has been classified as a possible human carcinogen and exposure through contaminated water, food, or occupational contact raises significant safety concerns. Laboratory-based instruments provide a robust methodology for the measurement of diuron, but there is an opportunity for electroanalytical based devices to provide an in-the-field approach that is comparable and, in some cases, can provide enhanced sensitivity. The low-cost and portable nature of electrochemical instruments allows one-site analysis, removing sample transportation and storage costs, and reducing the overall measurement time. In this perspective, we summarize recent advances in the measurement of diuron using electroanalytical methods, providing insights into the measurement of diuron using various sensing materials and electrochemical platforms. A wide range of electrode materials, such as carbon-based nanomaterials, metal nanoparticles, and molecularly imprinted polymers, have been explored to enhance sensitivity and selectivity in the measurement of diuron, and furthermore, we consider the use electrochemiluminescence and additive manufacturing. This overview highlights the role of material properties, electrode surface modification strategies, and signal amplification to enhance the electroanalytical detection of diuron, offering insights into current advancements and future directions in electrochemical sensing for environmental monitoring.

## Graphical abstract

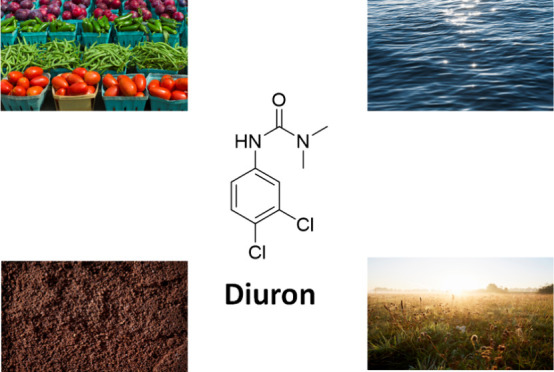

## Introduction to Diuron

Diuron (IUPAC name: *N*’-(3,4-dichlorophenyl)-*N*,*N*-dimethylurea) belongs to the phenylurea class of herbicides. Diuron is a white, odorless, crystalline solid with a melting point of 158–159 °C, boiling point of 180–190 °C, and water solubility of approximately 42 ppm (mg/L) at 25 °C; see Figure 1A for the chemical structure of diuron.^1^ Furthermore, the p*K*_a_ of diuron is 13.18 indicating that it is going to be in its protonated form (diuron). Diuron was introduced in 1954 by E. I. du Pont de Nemours & Company under the trademark “Karmex” and is mainly used as a nonselective systemic herbicide for general weed control in agricultural crop areas, (e.g., cereal, fruit, sugar cane) and as a soil sterilant in nonagricultural areas, (e.g., garden areas road sides, railway lines etc.).^1,2^ Diuron is also used to control algae (algaecide) by inhibiting photosynthesis, through blocking the electron transfer process in photosystem II.^3^ In mammals, diuron is metabolized by dealkylation of the urea methyl groups. Hydrolysis of diuron to 3,4-dichloroaniline and oxidation to 3,4-dichlorophenol as well as hydroxylation at carbon 2 and/or 6 of the benzene ring have also been reported.^1^ Diuron is metabolized to *N*-(3,4-dichlorophenyl)-urea in urine and it is also partially excreted unchanged in feces and urine.^1^ Diuron poses significant environmental risks, particularly to aquatic ecosystems, leading to regulatory restrictions in many countries. Diuron is persistent, with a half-life of 90–180 days in soil and it is toxic, with a classification as a possible human carcinogen by the U.S. Environmental Protection Agency, based on studies showing an increased incidence of bladder and kidney tumors in laboratory animals. Diuron may enter the human body through contaminated food or drinking water, particularly where it has leached into groundwater, or through occupational exposure during its handling or application. Chronic exposure has been linked to kidney and liver toxicity, including inflammation, enlargement, and altered enzyme activity in these organs, as well as cancer, endocrine disruption, and chronic toxicity. As such, concerns about long-term exposure and groundwater contamination have prompted heightened monitoring and tighter controls on its use water, soil, and food sources to minimize human exposure and protect public health.^2,4,5^

**Figure 1:**
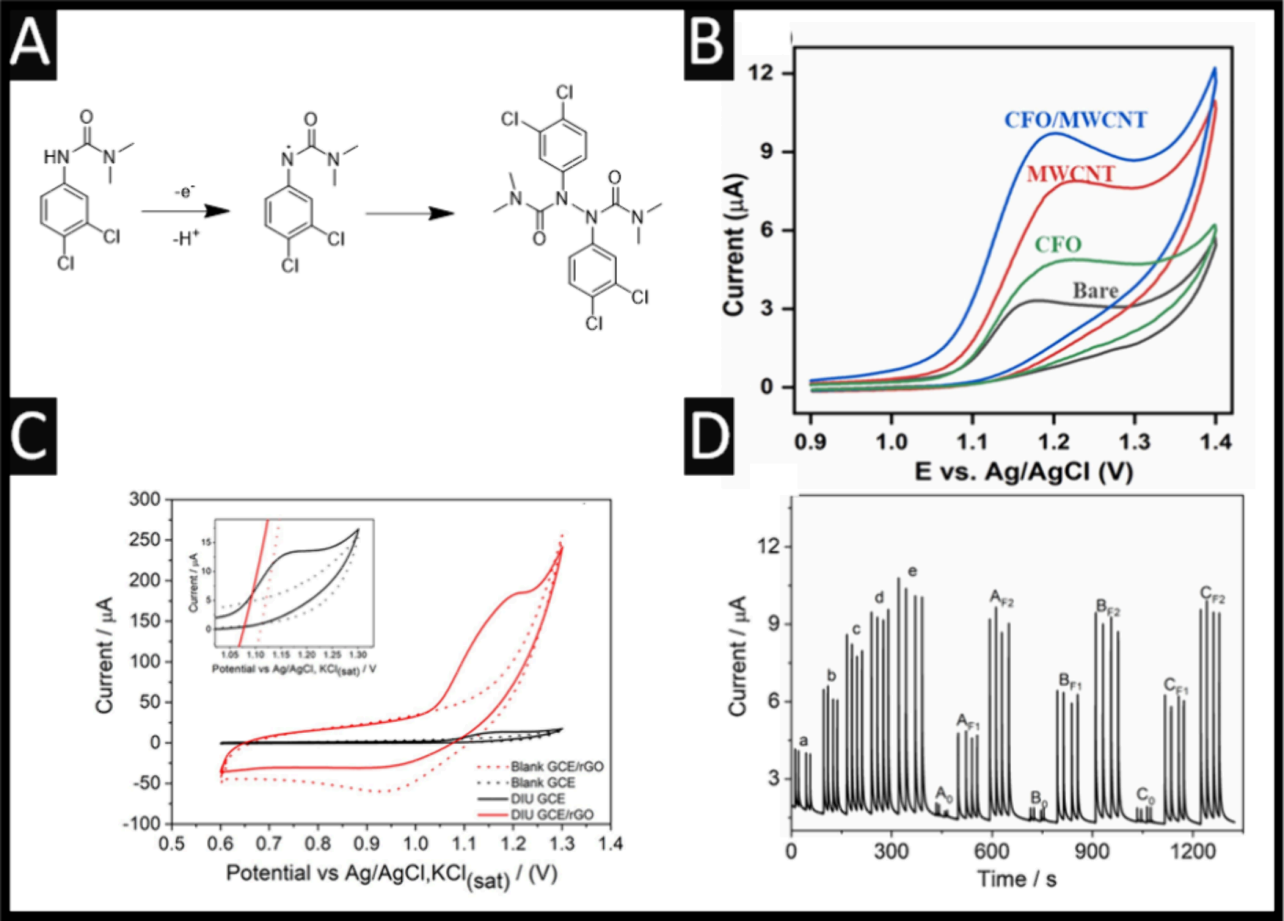
(A) The chemical structure of diuron and its electrochemical oxidation which shows that 2 molecules combine leading to the formation of free radical dimers. Figure adapted from reference 14. Copyright 2020 Elsevier. (B) Cyclic voltammograms of bare glassy carbon electrode, modified with CuFe_2_O_4_, MWCNT, and CuFe_2_O_4_@MWCNT. Parameters: 100 μM diuron; Scan rate: 50 mVs^–1^; pH 2. Figure reproduced from reference 38. Copyright 2025 Elsevier. (C) Cyclic voltammograms in the diuron (312.5 μM in pH 2) using a glassy carbon electrode and glassy carbon electrode–rGO composite electrode. (D) Amperogram for diuron using batch injection analysis using the tap water, orange and grape juices (A_0_, B_0_, and C_0_, respectively), spiked tap water (A_F1_, A_F2_), spiked orange juice (B_F1_, B_F2_), and spiked grape juice (C_F1_, C_F2_). Analytical curve: (a) 10, (b) 20, (c) 30, (d) 40, (e) and 50 μM; potential vs Ag/AgCl, KCl­(sat): + 1.2 V, injection volume: 75 μL, dispersing rate: 316.8 μL s^–1^. Figure reproduced from reference 26. Copyright 2022 Elsevier.

Diuron has been banned or heavily restricted in several countries due to its environmental persistence and toxicity to aquatic ecosystems. A recent review has provided an overview of water pollution by diuron,^6^ and readers who wish to measure diuron are advised to see Table 2 and 3 in the mentioned review article.^6^ In summary, the maximum permissible limits of diuron in drinking water is 100,000 ng L^–1^ in United States, 30,000 ng L^–1^ in Canada, while in Brazil, Japan and Australia is limited to 20,000 ng L^–1^. In the European Union, it is lower, at 100 ng L^–1^. It is notable that in the case of Mexico, Colombia, Brazil and Japan diuron has unrestricted use, whereas in United Kingdom, Nepal, Mozambique, Egypt, and Saudi Arabia, its use is prohibited.^6^

In the United States, diuron is not fully banned but is classified as a restricted-use pesticide with significant limitations due to environmental and health concerns.^7^ The U.S. Environmental Protection Agency (EPA), in its 2022 proposed interim decision, recommended canceling nearly all herbicidal uses of diuron, including its application on food and feed crops and nonagricultural sites such as roadsides and utility corridors.^7^ This decision was based on findings that diuron poses carcinogenic risks to humans through dietary and occupational exposure as well as significant ecological risks to birds, mammals, aquatic life, and plants. However, the U.S. EPA proposed retaining its use as a harvest aid on cotton and for specific nonherbicidal applications, such as in paints and residential ponds, with strict protective measures. While a final decision is pending, these proposed restrictions reflect the growing regulatory efforts to mitigate the risks associated with diuron in the U.S.^7^ These regulatory actions reflect growing global concern over the long-term environmental impacts of diuron, particularly its potential to leach into groundwater and disrupt aquatic ecosystems. Consequently, there is the need for the monitoring of diuron within soil, foods, and water sources to assess exposure levels, guide regulatory decisions, and protect both environmental and human health. Methods for the measurement of diuron include, but are not limited to gas chromatography–mass spectrometry,^8^ reversed-phase high-performance liquid chromatography coupled with atmospheric-pressure chemical-ionization mass spectrometry,^9^ capillary electrophoresis with electrochemiluminescence detection,^10^ infrared spectroscopy,^11^ and of course, electrochemistry-based techniques.

## Introduction to Electroanalysis

Electrochemistry, compared to laboratory-based instruments, is a low cost and simple to operate system that can provide rapid selectivity and sensitivity. Due to its ease of miniaturization and portability, it can be used to translate laboratory-based optimization into in situ measurements and provide on-site analysis. In this perspective, we summarize the development of electrochemical based sensors toward the measurement of diuron. Table 1 reports the various approaches for the measurement of diuron which are presented in order of year the paper was published, and one can observe the low limits of detection (LoD), vast linear ranges, sample media, preparation, and preconcentration are also presented. In this review, we first consider the use of electrode materials, fabrication techniques, biorecognition strategies, and detection modalities.

**Table 1:** An Overview of Approaches Reporting the Sensing of the Diuron[Table-fn t1fn2]

electrode	electrochemical technique	modification	linear range	limit of detection	sample media (preparation)	comments	reference
GCE	amperometric	*poly*-Ni(OH)TAPc	30–350 μM	0.3 μM	tap water (spiked, L-L extraction) and agricultural soil (spiked, S-L extraction)		^15^
GCE	amperometric	C_64_H_80_N_8_NiO_8_ complex	9.9 −150 μM	6.14 μM	river water (spiked, filtered, solvent-assisted L-L extraction); soil (spiked, filtered, S-L extraction)		^16^
GCE	Amperometric	GO-MWCNTs	9 μM - 0.38 mM	1.49 μM	well, lake, and irrigation ditch water (filtered, diluted, spiked)	simultaneous measurement of diuron and fenuron	^17^
CPE	SWV	MIP/MWCNTs-COOH	0.52 nM – 1.25 μM	9 nM	river water (spiked, filtered)	compared with HPLC	^18^
SPE	amperometric	acetylcholinesterase/AuNPs	80–1400 nM	50 nM	river water (spiked)		^19^
SPE	LSV	rGO–AuNP	0.17–4.29 μM	0.09 μM	lake and seawater (NS)		^20^
GCE	AdDPV	rGO-AuNPs/Nafion	1–100 nM	0.3 nM	orange juice, tea, mineral and tap water (spiked)	compared with HPLC	^21^
GCE	DPV	SiO_2_@AuNPs	0.2–55 μM	52 nM	tomato, spinach and cucumber (S-L extraction, spiked)	compared with LC-MS/MS	^22^
BDD	DPV	cathodically treated; preconcentration using a solid phase extraction	1–9 μM	0.035 μM	solid phase extraction shown. Lake and well water ((acidified, filtered, spiked)	simultaneous sensing of diuron, 2,4- dichlorophenoxyacetic acid and tebuthiuron.; compared with HPLC-DAD	^13^
Pencil	DPV	MIP	10–500 μM	43.43 μM	tap and irrigation water (NS)		^23^
GCE	DPV	MWCNT-COOH	0.21–2.5 μM	68 nM	seawater (filtered, spiked)		^24^
CPE	SWV	nanocrystalline cellulose	4.2–47 μM	0.35 μM	soil (S-L extraction, diluted, spiked)		^25^
GCE	amperometric	rGO	5–50 μM	0.36 μM	tap water, grape, and orange whole juice (diluted, spiked)	BIA analysis	^26^
GCE	DPV	MWCNTs-CS@NGQDs	0.3–51.5 μM	0.2 μM	river water (filtered, diluted, spiked), soil (crushed, S-L extraction, filtered, diluted spiked)	compared to UPLC-MS/MS	^27^
CPE	SWV	zinc oxide NPs	1.3–7.7 μM; 8.6–30 μM	0.22 μM	soil (S-L extraction, spiked) and river water (centrifuged, dilution, spiked)		^28^
GCE	DPV	tungsten oxynitride nanosheets	0.01–764.4 μM	5.5 nm	grape, orange juice, tap, and river water (diluted, spiked)		^29^
GCE	DPV	Co, Mn oxides nanoparticles-functionalized boron nitride	0.01–419 μM; 569–1770 μM	13 nM	river and tap water (centrifugation, dilution, spiked)		^30^
CPE	DPV	octa(aminopropyl)silsesquioxane/Prussian blue nanoparticles	10–900 nM	4.96 nM	tap water (spiked)		^31^
GCE	SWV		38.5–115 nM	0.2 nM	river water (dilution, filtered, spiked)		^12^
GCE	DPV	MIPs/Gold nanocages/NH_2_-rGO	4.3–42 μM	18 nM	cotton (L-L extraction, spiked), soil (S-L extraction followed by salt-induced L-L phase separation, spiked)	compared to HPLC-UV	^32^
GCE	ECL	MIPs/AuNCs/TEA	21 nM – 2.1 μM	9.7 pM	canal water (filtered, spiked)	compared to HPLC-MS	^33^
ABS waste from 3D-printing/graphite	SWV		0.25–2.5 μM; 5–20 μM	68.9 nM	sugar case juice and *cachaça* (dilution, spiked)		^34^
GCE	DPV	bismuth oxide microplates/GO	0.1–631 μM	0.751 μM	carrot, potato and pond water (NS)		^35^
CPE	SWV	holmium oxide NPs (Ho_2_O_3_)	0.25–200 μM	0.03 μM	strawberry, apple juice, tap water (L-L extraction, spiked).	compared with UV–vis	^36^
SPE	DPV	MWCNT	1.07–7.51 μM	0.112 μM	seawater, grape juice (no-pretreatment, spiked).		^37^
GCE	DPV	CuFe_2_O_4_ NPs@MWCNT	0.01–180 μM	0.06 μM	carrot, cabbage, cucumber, orange (S-L extraction, spiked).	compared to HPLC	^38^

a Key: AuNCs: gold nanoclusters; AuNP: gold nanoparticles; BDD: boron-doped diamond electrode; BIA: Batch Injection Analysis CPE: carbon paste electrode; DPV: differential pulse voltammetry; ECL: Electrochemiluminescence; GCE: glassy carbon electrode; GO: graphene oxide; HPLC: High-Performance Liquid Chromatography; L-L: liquid–liquid extraction; LSV: linear sweep voltammogram; MIPS: molecular imprinting polymers; MWCNTs: multiwalled carbon nanotubes; MWCNTs-CS: chitosan-encapsulated multiwalled carbon nanotubes; NGQDs; nitrogen-doped graphene quantum dots; NPs: nanoparticles; NS; not stated; rGO–AuNP: reduced graphene oxide–gold nanoparticle; rGO: reduced graphene oxide; S-L: solid–liquid extraction; SWV: Square-wave voltammetry; TEA: triethylamine; UPLC-MS/MS: ultraperformance liquid chromatography-tandem mass spectrometry.

## Electrode Materials for the Sensing of Diuron

The use of bare electrodes for the sensing of diuron has been explored, predominantly using carbon-based materials. For example, using a bare glassy carbon electrode has provided a linear response of 38.5–115 nM with a low LoD of 0.2 nM, which was shown to be successful in the evaluation of diuron in river water close to sugar cane cultivation in the state of Paraíba, Brazil.^12^ Other work has reported the simultaneous determination of diuron, 2,4-dichlorophenoxyacetic acid and tebuthiuron using a cathodically pretreated boron-doped diamond electrode in conjunction with differential pulse voltammetry has been reported.^13^ The authors used a solid phase extraction approach, with polyvinylimizadole cross-linked with trimethylolpropanetrimethacrylate as the adsorbent, where a multiresidue solution is added. The elution is performed with ethanol, and the eluate was then evaporated to dryness on a hot plate and redissolved into 0.1 M sulfuric acid. The boron-doped diamond electrode is anodically pretreated by applying 0.5 A cm^–2^ (3.0 V) for 30 s and cathodically pretreated by applying −0.5 A cm^–2^ (−2.0 V) for 120 s, in a 0.5 mol L^–1^ sulfuric acid solution. The authors infer that the boron-doped diamond electrode surface is predominantly hydrogen terminated,^13^ but further work is needed to quantify the specific surface terminations resulting from anodic and cathodic treatments. This approach took forward the cathodically pretreated boron-doped diamond, where they reported that the cathodic pretreatment provides better performance in terms of the peak height/sensor response. The authors validated their sensor measuring diuron and associated herbicides, 2,4- dichlorophenoxyacetic acid and tebuthiuron spiked in lake and well water, which is compared against high-performance liquid chromatography providing recoveries in the range of 96–104%. These results were shown to be statistically validated.^13^ Importantly, this study shows that the simultaneous determination of multiresidues can really be used in place of laboratory based instruments. The sensing of diuron can be achieved on other bare electrodes such as graphite and screen-printed carbon electrodes, but these are limited due to the low sensitivity, excessive overpotential, and electrode fouling in diuron sensing. The electrochemical mechanism of diuron is shown in Figure 1A, where diuron is electrochemically oxidized and it loses one electron and one proton, leading to the formation of free radical dimers via amino group interactions.^14^ To overcome these limitations associated with using bare electrochemical platforms, authors direct their research to the development and use of micro- and nano- sized materials.

Multiwalled carbon nanotubes (MWCNTs) are routinely employed in the modification of electrochemical surfaces due to their reported structural, mechanical, electronic, and electrochemical properties. These include high surface area and the presence of numerous active sites, such as edge-plane-like sites/defects and oxygen functional groups.^39^ Such modification of electrode surfaces typically enhances the electroanalytical performance toward specific analytes by increasing the density of active sites and improves electron transfer. However, the application of MWCNTs introduces complexities, since the porosity provides an ill-defined mass transport regime both to and within the porous layer, which can be dominated by thin-layer effects, potentially creating the illusion of electrochemical reversibility. From inspection of Table 1, one can see that MWCNTs are regularly used with other nanobased materials. This approach using MWCNTs is exemplified within Figure 1B, where one can observe the electrochemical oxidation of diuron occurs at the bare glassy carbon electrode at +1.15 V (Ag/AgCl), which is transformed into a larger signal with the modification of CuFe_2_O_4_, MWCNTs, and CuFe_2_O_4_@MWCNT, of which the combination of CuFe_2_O_4_ with MWCNTs results in the largest response at +1.25 V (Ag/AgCl).^38^ This increase in the signal originates from the addition of the MWCNTs, which gives rise to a larger electrochemical area. Another notable approach has been reported by Alves and co-workers who presents a novel, fast, and sensitive method for detecting diuron using a batch injection analysis system with amperometric detection coupled to a glassy carbon electrode modified with reduced graphene oxide.^26^ The use of batch injection analysis holds the potential to electroanalytically detect diuron (typically +1.2 V vs Ag/AgCl). This is achieved through injections of increasing concentration of diuron being rapidly oxidized, and the resulting increase in current being proportional to the analyte concentration. This current is measured over time, producing sharp transient signals. Batch injection analysis systems offer advantages such as high throughput, minimal sample and reagent consumption, and simple instrumentation, making them well-suited for rapid and cost-effective analyses. As shown in Figure 1C, one can observe the electrochemical oxidation of diuron at a bare glassy carbon electrode, which shows an electrochemically irreversible signal at +1.15 V (vs Ag/AgCl), which is also observed at a similar potential of +1.16 V (vs Ag/AgCl) using a reduced graphene oxide modified glassy carbon electrode surface.^26^ The use of reduced graphene oxide results in a 7.6 fold increase of the current response, where possibly, the reduced graphene oxide offers a higher surface roughness and therefore more electroanalytical sites;^26^ this sensor demonstrated an LoD of 0.36 μM.

To optimize the analytical conditions, the authors investigated the effects of pH, applied potential, injection volume, and dispersion rate. The best performance was obtained at pH 2.0 and an applied potential of +1.2 V (vs Ag/AgCl). As can be seen in Figure 1D, the amperograms for the diuron sensing are shown where a, b, c, d, and e are the concentrations of 10, 20, 30, 40, and 50 μM respectively, providing the calibration curve. This is then extended to the measurement of spiked tap water, grape, and orange whole juices reporting good repeatability (RSD < 3.7%), and recovery rates between 80.8% and 105.5%. This method achieved high analytical throughput, 150 samples/hour and required only minimal sample preparation (simple dilution in buffer), making it well-suited for routine and on-site environmental or food safety testing. In comparison to other electroanalytical techniques for diuron detection, this method provides a facile performance making this approach a practical alternative to more complex or time-consuming methods such as gas and liquid chromatography.

A composite has been fabricated using chitosan-encapsulated MWCNTs (MWCNTs-CS) combined with nitrogen-doped graphene quantum dots (NGQDs), where the NGQDs are prepared by high temperature pyrolysis providing a composite of MWCNTs-CS@NGQDs.^27^ This is examined toward the inner-sphere redox probe [Fe­(CN)_6_]^3–/4–^, which reports that for a bare glassy carbon electrode, the peak-to-peak separation (Δ*E*_p_) was 108 mV. The anodic peak current of 57 μA was increased to 99 μA and the Δ*E*_p_ reduced to 96 mV using the MWCNTs-CS@NGQDs. The improvement is attributed to a large electrochemical area and improved electron transport capability,^27^ using the numerous edge plane sites/defects on the MWCNTs. The authors then consider the sensing of diuron, where the bare glassy carbon gave rise to the electrochemical oxidation at ∼1.2 V (vs Ag/AgCl), which in the presence of the MWCNTs-CS@NGQDs gave rise to a large and quantifiable signal but still occurred at the same oxidation potential. This is attributed to the beneficial edge plane sites/defects and functional groups (C/O, etc.) on the MWCNTs that provide adsorption capabilities. This composite is shown to measure diuron over the range of 0.3–51.5 μM, with a LoD of 0.2 μM. Interferents have been explored, where the concentration of the interfering ions is 100 times higher than that of diuron. The detection in the presence of Ca^2+^, Mg^2+^, K^+^, Cu^2+^, SO_4_^2–^, Cl^–^, acetamidine, imidacloprid, and ethephon were investigated, which had little effect on the sensor’s performance. This sensor has merit in the sensing of spiked diuron in river water and soil, where, in the former sample, river water is collected from the Beijing-Hangzhou Grand Canal in Zhenjiang, China, and is filtered through a 0.22 μM filter membrane before being diluted 20 times with pH 2 buffer solution. In the case of the former sample, solid was collected from the Xinjiang region where 5 g of crushed soil samples were added into 5 mL of water and 10 mL of acetonitrile, then extracted by shaking for 10 min. Next, 4 g of NaCl was introduced to induce the separation of aqueous phase and the organic phase in 5 min. The soil supernatant is filtered through a 0.22 μm filter membrane, where this is again diluted 20 times with pH 2 buffer solution. This sensor is able to measure spiked diuron in river water and soil reporting recoveries across the range of 99.4–104.0% and 90.0–94.6%, respectively, which are directly compared against ultrahigh performance liquid chromatography coupled with tandem mass spectrometry, providing evidence of the consistency between the two methodologies.^27^ The use of graphene oxide–MWCNT composites for the sensitive determination of diuron and fenuron using amperometry (using a rotating disc electrode) have been reported, which provides a very large linear range of 9 μM–0.38 mM and LoD of 1.49 μM. Fenuron is a phenyl urea class herbicide and this is the first report of its sensing, where the peaks are well resolved from each other allowing the authors to measure both compounds.^17^ This sensor is shown to be successful in the measurements of spiked well, lake, and irrigation ditch water, reporting recoveries of 95.0–103.7%.^17^ The use of GO-MWCNTs are reported to form a three-dimensionally arranged hierarchical structure, which can offer the highest edge density per unit normal area and has found application in various areas.^40–42^ This composite is simply synthesized using graphite, which is subjected to the Hummers method to produce graphene oxide. The MWCNTs are added in a ratio to the GO of 2:1, which are subjected to ultrasound for 2 h to realize the GO-MWCNTs. These are shown to have thickness of 1–2 nm and have a range of surface oxygenated functional groups. These are drop casted onto the glassy carbon electrochemical platform using GO-MWCNTs dispersed into water, and for comparison, these are also dispersed in DMF. These were assessed toward the measurement of diuron, where the authors report a LoD of 1.49 μM for the water-based method. The authors show that in the case of the bare electrode and a modified GO, no signal is observed but in the presence of GO-MWCNTs and DMF-GO-MWCNTs, signals are observed at a potential of +0.80 V and +0.88 V (Ag/AgCl), respectively. This is attributed to^17^ (1) GO acts as a dispersant for MWCNTs, which facilitates a higher percentage of MWCNT dispersion than DMF-MWCNTs, (2) the formation of a 3D hierarchical GO–MWCNT network provides the highest edge density per unit nominal area, and (3) the presence of GO avoids aggregation of MWCNTs and makes the dispersion stable for more than 6 months. The voltammetric peak shows thin-layer behavior but that said, the authors have used this sensor for diuron and fenuron, another herbicide, and have applied it successfully to well, lake and irrigation ditch water.^17^ Other work has reported the use of MWCNT-COOH who observed the electrochemical oxidation of diuron at +1.14 V (Ag/AgCl) using the bare glassy carbon electrode which shifted to +1.10 V (Ag/AgCl), which was attributed to the electrocatalytic effects of functionalized MWCNTs.^24^ Returning to the start of this paragraph, one might consider the term used to describe MWCNTs as “electrocatalytic activity”, where in all cases, an ensemble of MWCNTs shows mixed mass transport behavior complicating and precluding the elucidation of their catalytic behavior. This has been shown to be overcome by the use of single nanoimpact electrochemistry;^43^ researchers should adopt this approach before labeling their MWCNTs as being “electrocatalytic”.

One of the earliest papers for the detection of diuron reported the use of polymerized nickel tetraamino-phthalocyanine (NiTAPc), containing O–Ni–O bridges. Represented as poly-Ni­(OH)­TAPc modified glassy carbon electrodes, they were used for the sensing of diuron, exploring the sensor in spiked tap water and soil using liquid–liquid and solid–liquid extraction, respectively.^15^ The use of phthalocyanine has reported electrocatalytic activity, which results in a reduced overpotential of 60 mV and increased electrochemical area; the mechanism is shown in Scheme 1:

**Scheme 1:**
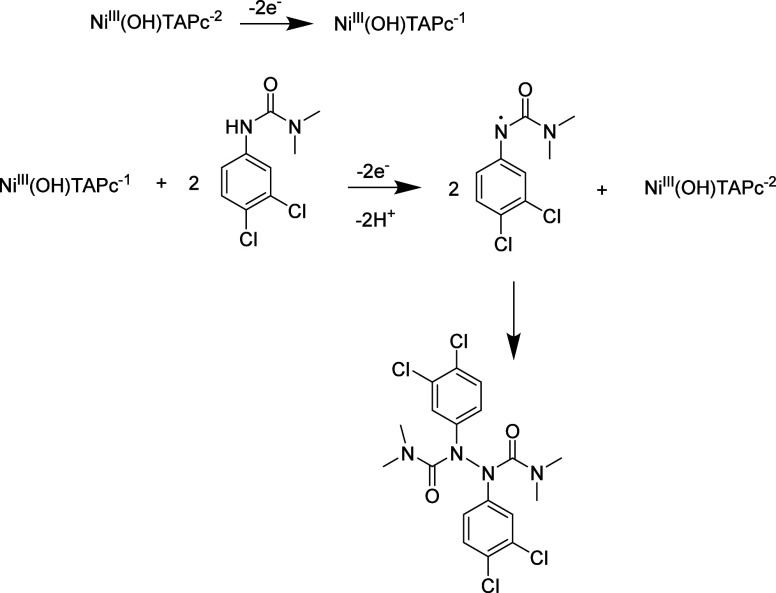
Electrocatalysis of Diruon Using a poly-Ni­(OH)­TAPc-Modified Glassy Carbon Electrodes

Others have studied a biomimetic based sensor using nickel­(II) 1,4,8,11,15,18,22,25-octabutoxy-29*H*,31*H*-phthalocyanine (C_64_H_80_N_8_NiO_8_ complex) based on the P450 enzymes, which are known to be involved in the metabolism of many drugs, steroids, and carcinogens in living organisms.^16^ In their approach, the authors showed that the complex, incorporated into a bulk modified carbon paste electrode, gave rise to a 6.4-fold increase in sensitivity compared to a bare carbon paste electrode. This was shown to be applicable for the sensing of diuron in spiked river water and soil samples, providing recoveries across the range of 94–106%.^16^

One can observe from inspection of Table 1 that metal/metal oxide nanoparticles and related structures are reported for the sensing of diuron either used alone or made into a composite structure.^19–22,28–30,32,33,36,38,44^ Metal/metal oxide nanoparticles are commonly used due to their nanoscale dimensions, which provide a high surface area-to-volume ratio, exposing more active sites for electrochemical reactions and significantly enhancing reaction kinetics by providing electrocatalytic properties. One interesting aspect that some forget to state in their research is that an electrochemical surface, which is decorated with nanoparticles, is similar to that obtained if one had used a complete electrode, e.g., a film or a solid electrode of the same material. This is due to heavy diffusional overlap occurring at each nanoparticle, which is a unique property where a nanoparticle array yields a similar amount of electrolytic depletion to a macroelectrode of the same total area. As such, minimal amounts of expensive nanoparticles can be used to offer a maximal electroanalytical output over that of a solid electrode with significant cost savings.^45,46^ For example, zinc oxide nanoparticles are shown to provide an LoD of 0.22 μM and two linear ranges from 1.3–30 μM.^28^ The zinc oxide nanoparticles are synthesized by a hydrothermal methodology, which results in an average particle size of 140 nm. One can observe the morphology which in Figure 2A, where the particles are agglomerates providing a large surface area and high surface energy.^28^ These zinc oxide nanoparticles are incorporated into a carbon paste electrode by simply mixing the nanoparticles with graphite powder and silicone oil. In model solutions, pH 7.4, one can observe that a large signal is obtained using the zinc oxide nanoparticle carbon paste electrode; see Figure 2Aiii. This is attributed to the increased surface are of the use of the zinc oxide nanoparticles.^28^

**Figure 2:**
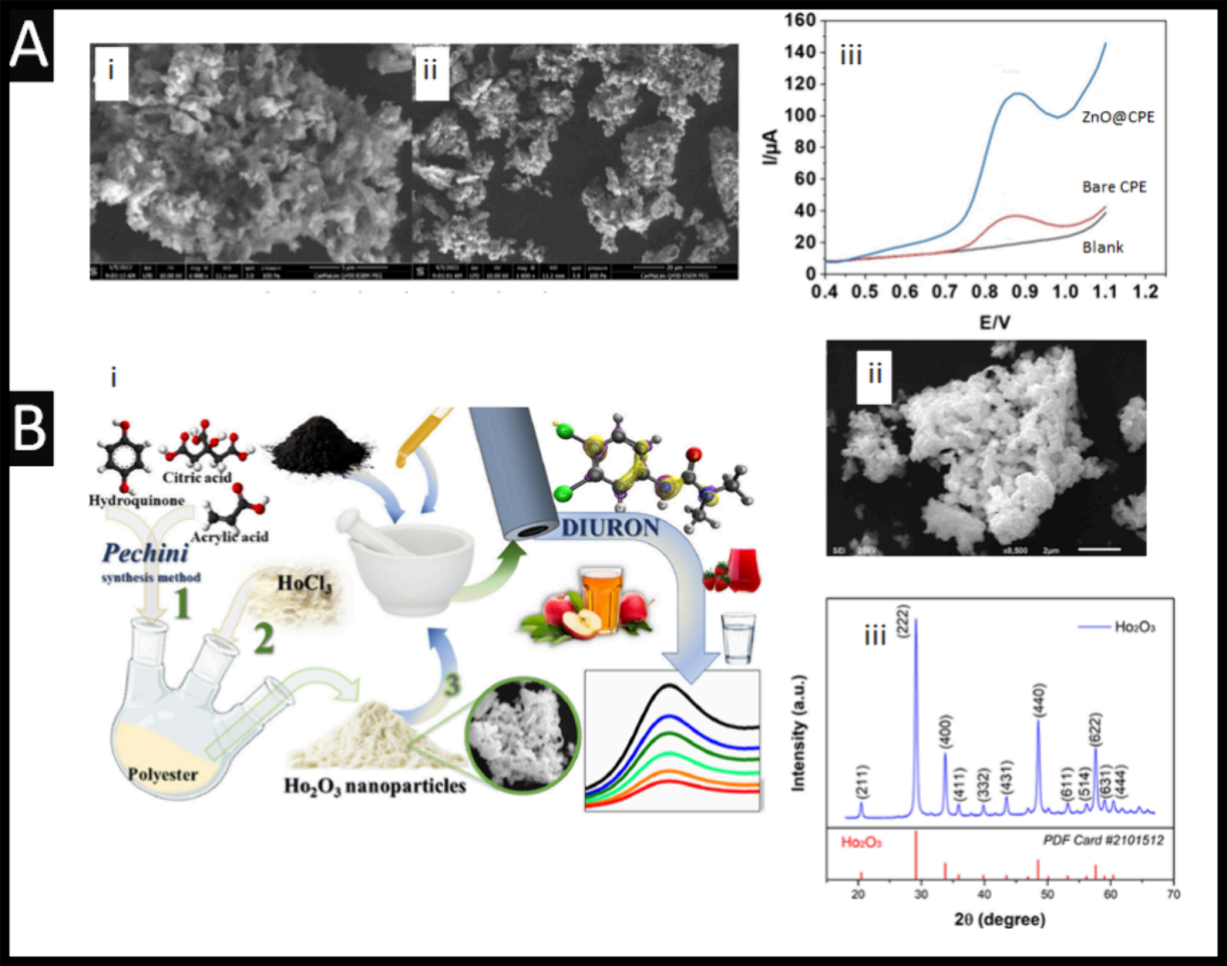
(A) (i and ii): Scanning electron microscopy images of the zinc oxide nanoparticles. (iii) Measurement of diuron obtained using a blank, bare carbon paste electrode (CPE and a zinc oxide nanoparticles carbon paste electrode (ZnO@CPE) using square-wave voltammetry. Parameters: pH 7.4; 9 mg/L diuron. Figure reproduced from reference 28. Copyright 2023 Wiley. (B) (i) An overview of the modified Pechini method for producing Ho_2_O_3_ nanoparticles, which are then bulk modified with a carbon paste electrode. (ii) Scanning electron microscopy image of the Ho_2_O_3_ nanoparticles. (iii) X-ray diffractogram of Ho_2_O_3_ nanoparticles. Figures reproduced from reference 36. Copyright 2025 Elsevier.

The authors explored this sensor further by evaluating the signal of the diuron in the presence of the following species: K^+^, Na^+^, Fe^3+^, Al^3+^, NO^3–^, SO_4_^2–^, and Cl^–^, where the authors state that none of these are up to 17 μM, resulting in significant interference.^28^ This sensor was explored in the measurement of diuron in river water and soil samples. The river water samples were collected, centrifuged, filtered, and then diluted by a factor of 10 using a pH 8 buffer solution followed by being spiked with diuron. In the case of the soil, it is first spiked with diuron, which is dissolved into methanol. Next, the sample is shaken for 2 h followed by centrifugation and filtration. The filtrate obtained was evaporated and dried at 40 °C in an oven, then dissolved in 1 mL of methanol, and diluted by a factor of 10 using a pH 8 buffer solution.^28^ Using the standard addition protocol, the authors performed recovery experiments, which ranged from 92–108%. The authors should consider expanding their study by comparing to laboratory-based methods and examining real samples (unspiked).

The use of a reduced graphene oxide-gold nanoparticles-Nafion composite film constructed upon a glassy carbon electrode has been reported providing a low linear range of 1–100 nM and LoD of 0.3 nM.^21^ Meanwhile, the exact average size of the gold nanoparticles is not explicitly stated in the text, from SEM images and the corresponding histogram presented,^21^ indicating that the gold nanoparticles are uniformly dispersed. From this data it is reasonable to infer that the gold nanoparticles have an average diameter in the range of approximately 10–30 nm, which is optimal for high surface area and potential electrocatalytic activity. This sensor is attributed to the synergy of using reduced graphene oxide, gold nanoparticles, and the use of a high cation-exchange capacity of Nafion. This helps adsorb diuron from the bulk solution to the increased electrode surface and high conductivity of reduced graphene oxide and gold nanoparticles, which has led to the author using adsorptive differential potential voltammetry.^21^ This sensor is applied toward the sensing of spiked diuron in orange juice, mineral, and tap water samples reporting recoveries across the range of 90–110%. Also, diuron was determined in tea samples using this sensor, showing good agreement with high performance liquid chromatography.^21^ This sensor can increase its adsorptive time even further to reduce the LoD.

Another example reports the synthesis of holmium oxide (Ho_2_O_3_) nanoparticles (diameter of 26 ± 5 nm) using a modified Pechini method. The nanoparticles are applied in the development of a carbon paste electrode (CPE/Ho_2_O_3_) for electrochemical sensing of diuron.^36^ The authors used a modified Pechini method,^47^ shown in Figure 2i, involving the formation of a polymeric precursor by complexing metal ions with hydroxycarboxylic acids (such as citric acid). This is followed by polyesterification with a polyhydroxy alcohol (e.g., ethylene glycol or acrylic acid). In this work, citric acid and acrylic acid were used along with hydroquinone to initiate polymerization followed by the addition of Ho^3^^+^ ions. The resulting high-viscosity resin was dried and subjected to pyrolysis at 450 °C for 4 h and then calcined at 900 °C for 4 h to yield crystalline Ho_2_O_3_ nanoparticles. Figure 2Bii shows an SEM image of the Ho_2_O_3_ nanoparticles, where SEM-EDX confirmed the elemental composition of 81.3 wt % of Ho and 18.2 wt % of O; the difference of 0.5 wt % is due to impurities introduced during the synthesis.^36^ Also shown in Figure 2Biii is the X-ray diffractogram, which shows a body-centered cubic (bcc) structure with I*a* (No. 206) space group symmetry.^36^ The standard diffractogram of Ho_2_O_3_ (PDF #2,101,512) is also shown. This sensor is explored toward diuron, reporting a low LoD of 0.03 μM, and was explored toward interferents, namely, K^+^, Mg^2^^+^, Ca^2^^+^, NO_3_^–^, Cl^–^, and SO_4_^2–^, glucose, vitamin B1, and vitamin C, carbofuran, carbendazim, glyphosate, bentazone, and linuron. Each interferent was introduced at a concentration 100 times higher than that of diuron (10 μM) where the sensor demonstrated minimal interference, with current changes below 8.5%, confirming the sensor’s excellent selectivity and its suitability for reliable detection of diuron in complex environmental and food matrices. The use of Ho_2_O_3_ provided an electrocatalytic activity though large surface area and low *R*_ct_ (charge transfer resistance).^36^ This sensor is shown to be beneficial for the measurement of spiked diuron in strawberry, apple juice, and tap water, which are spiked at 5, 10, and 20 μM and using calibration curves, the diuron concentration is determined and compared with UV–vis independent analysis, which provides a close match.^36^

Other work has reported the fabrication of gold nanoparticles, with an average size of 6 nm immobilized onto SiO_2_ nanoparticles with a diameter of 95 nm.^22^ First, uniform SiO_2_ nanoparticles were synthesized by using a modified Stöber method. Ammonia was mixed with ethanol and sonicated briefly. Then, a mixture of ethanol and TEOS was added slowly under stirring. The solution was heated and maintained at an elevated temperature for several hours. After washing and drying, the particles were annealed at high temperature. Next, the SiO_2_ particles were functionalized with amino groups. The particles were dispersed in isopropanol followed by the slow addition of APTES. After ultrasonic treatment, the mixture was refluxed for several hours. The product was separated by centrifugation, washed, and dried. Next, citrate-coated gold nanoparticles are synthesized, where a solution of gold precursor and sodium citrate was prepared in deionized water. A reducing agent was then added, and the solution was stirred briefly. The resulting gold nanoparticles were collected by centrifugation and were washed. Finally, the gold nanoparticles were attached to the amino-modified SiO_2_ particles. The SiO_2_ particles were stirred in deionized water followed by the addition of the gold nanoparticles solution. After further stirring, the resulting SiO_2_@gold nanoparticles were washed and dried under a vacuum. The powder was redispersed in water using sonication to form a uniform suspension.^22^ This sensor is shown to be viable for the measurement in tomato, spinach, and cucumber, where each sample is reduced in size via grinding. Methanol is added to the sample and stirred for 20 min, after which, the sample is centrifuged, and the supernatant is collected. A small sample (15 μL) is mixed with a pH 2.5 buffer solution. Using the standard addition method, the authors found that in the case of spinach, diuron is absent or potentially below the LoD, but for tomato and cucumber, diuron is present at levels of 4.4 and 2.5 μM, respectively, which is compared with LC-MS/MS and shows close agreement. This sensor shows promise for the routine detection of diuron in real vegetable samples and can be adapted for field deployment, offering a low-cost but yet sensitive approach for rapid analysis.

## Fabrication Techniques

3D printing or additive manufacturing is increasingly used in electrochemistry to enable the rapid and precise fabrication of complex, customized components such as electrochemical based sensors.^48–51^ Additive manufacturing offers unique advantages for the fabrication of electrochemical sensors compared with conventional manufacturing strategies. One of the key benefits is the ability to design electrodes with complex and creative geometries, which opens up new possibilities.^52^ Among the various additive manufacturing techniques, fused filament fabrication (FFF) has been by far the most widely used for producing electrochemical sensors, where its popularity is due to the versatility and simplicity of the FFF process, as well as the wide range of sustainable materials that can be achieved either through the use of commercially available or the design and fabrication of bespoke filaments.^48–51^ For example, Santos Oliveira et al. reported a composite material derived from recycled ABS filaments for 3D printing/additive manufacturing.^34^ As shown in Figure 3A, one can see that the authors have used ABS to print out their working electrode support, which is then used to form a conductive paste on recycled ABS, using ABS waste generated during the printing of the supports. The authors report that they take the ABS waste and cut it down, and acetone and chloroform are added in a 3:1 ratio, which is heated to 70 °C and stirred constantly for 20 min in a reflux system. After complete dissolution, different fractions of graphite were added to study ratios of ABS and graphite ranging from 70:30 to 50:50 (% w/w) where the authors reported that the 60:40 ratio provides the most desirable electrochemical response.^34^

**Figure 3:**
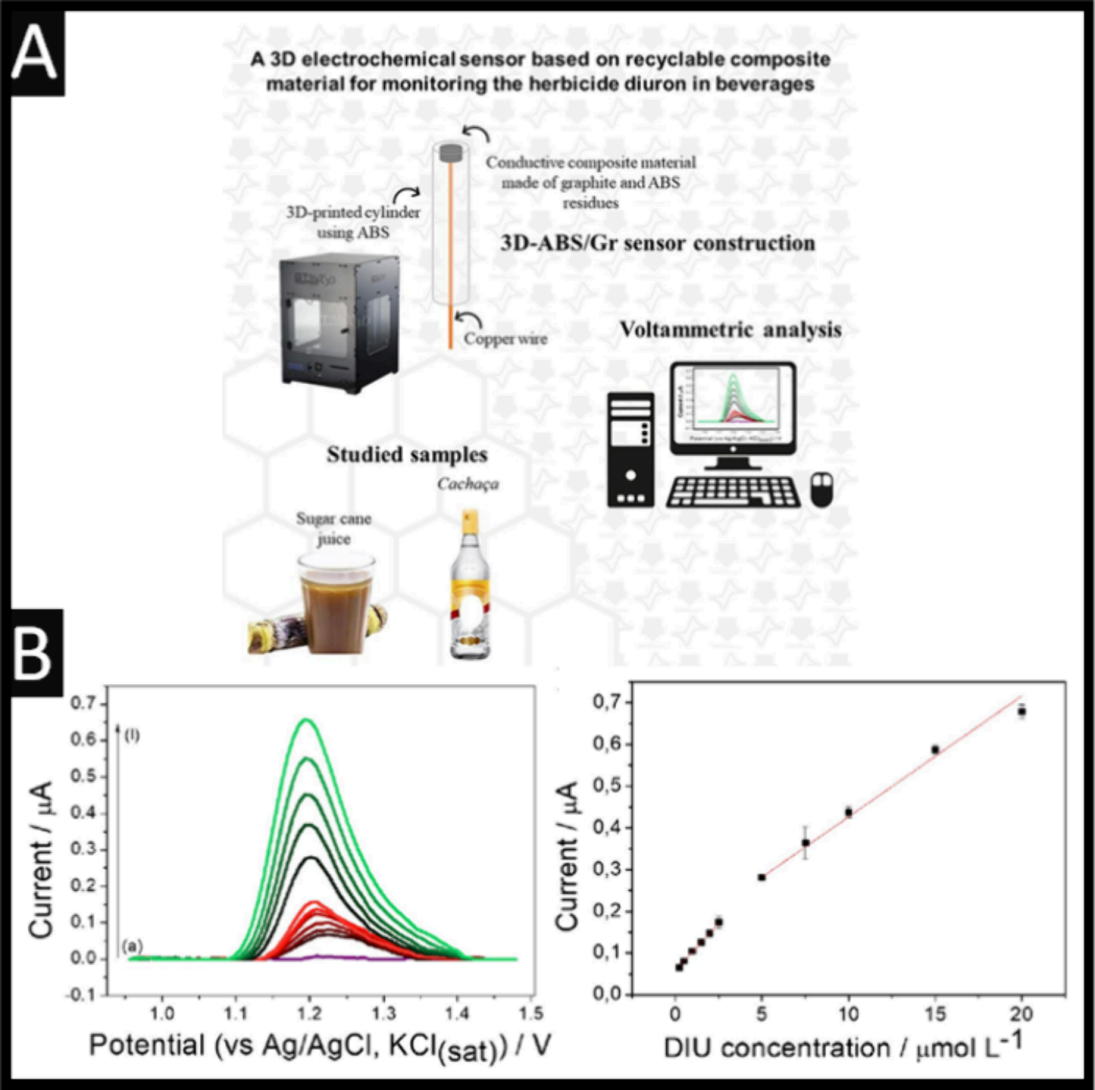
(A) An overview of the approach using 3D-printing/additive manufacturing to produce a sensor for the measurement of diuron in sugar case juice and *cachaça*. (B) Square-wave voltammograms were used for the sensing of diuron and associated analytical curve. Parameters: pH 2; amplitude of 40 mV, step potential of 8 mV, and frequency of 50 Hz. Figure reproduced from reference 34. Copyright 2024 Elsevier.

This sensor allows the measurement of diuron across two linear ranges of 0.25–2.5 and 5–20 μM and produced an LoD of 68.9 nM; see Figure 3B. The authors explored 10 replicates of the sensors reporting a 3.1% RSD and investigated the influence of fructose, glucose, sucrose, glyphosate, and dichlorophenoxyacetic acid using a concentration ratio of 1:1 with diuron. Using a 25 μM concentration, the electrochemical signal varied up to 7.3%, demonstrating selectivity for the measurement of diuron. Last, this sensor is shown to be used for the measurement of sugar case juice and *cachaça*, which are simply diluted with a supporting electrolyte 50 times, where the recovery rate ranged from 99.8–110.8%. This approach has useful benefits to realize low-cost based electrochemical sensors, which others should follow. Other fabrication techniques include the fabrication of screen-printed electrodes.^19,20,37^ Screen-printed sensors have become an essential platform due to their low cost, portability, ease of mass production, and compatibility with a wide range of surface modifications. These sensors typically consist of a working, reference, and counter electrode printed onto a flexible substrate using conductive inks, allowing for customizable geometries and scalable fabrication. Screen-printed sensors have been widely employed as platforms for the immobilization of nanomaterials, such as reduced graphene oxide–gold nanoparticle (rGO–AuNP) composites, acetylcholinesterase–gold nanoparticle conjugates, and MWCNTs.^19,20,37^ More recently,^37^ a low-cost, scalable fabrication of homemade screen-printed electrodes using graphite and alkyd resin conductive inks for the sensing of diuron in environmental and food samples has been reported. Optimization of ink composition and inclusion of toluene as a solvent significantly enhanced electrode performance by improving graphite dispersion, increasing electroactive area, and reducing charge transfer resistance.^37^ The optimal electrode (screen-printed electrodes with 50% alkyd resin, 50% graphite, and 1150 μL of toluene) was further modified with MWCNTs, yielding a sensitive and selective sensor with a low LoD of 0.112 μM. The sensor was shown to be useful for the sensing of spiked diuron in seawater and grape juice, which are simply diluted reporting a 97.1%–116% and 88.2%–11% recoveries, respectively. The authors noted that the electrochemical oxidation of diuron is reduced from +0.82 to +0.77 V (pseudo silver reference) in the presence of MWCNTs, which is attributed to the “enhanced electrocatalytic activity”^37^ of the MWCNTs. The use of MWCNTs are reported to prevent blockage of the electrode by the dimeric diuron species,^37,53^ but please see Figure 1A for an overview of the electrochemical mechanism who have used electrochemical coupled with mass spectrometry.^14^

This approach enables the translation of laboratory-based sensor technologies into field-deployable devices, supporting portable, real-time analysis in environmental and industrial settings.

## Biorecognition Strategies

Molecularly imprinted polymer (MIP)-based sensors have been reported.^18,23,32,33^ For example, a sensitive and selective electrochemical sensor for the detection of 3,4-dichloroaniline has been reported.^54^ Diuron is applied as a, herbicide which is biodegraded to 3,4-dichloroaniline and exhibits a higher toxicity.^2^ This sensor is based on MIPs synthesized on magnetic Fe_3_O_4_ nanoparticles with allyl alcohol as the functional monomer. The Fe_3_O_4_/MIP sensor was synthesized via a core–shell strategy integrating magnetic nanoparticles and molecular imprinting; see Figure 4A. Initially, Fe_3_O_4_ nanoparticles were prepared by coprecipitating Fe^2^^+^ and Fe^3^^+^ salts in an aqueous medium with NH_4_OH under nitrogen at 80 °C followed by magnetic separation and drying. These nanoparticles were then coated with silica using tetraethoxysilane and functionalized with 3-methacryloxypropyltrimethoxysilane to introduce polymerizable vinyl groups. For imprinting, 3,4-dichloroaniline was used as the template molecule, allyl alcohol was selected as the functional monomer (based on computational binding affinity), and ethylene glycol dimethacrylate served as the cross-linker. The monomer–template complex was preassembled in ethanol, then combined with the modified Fe_3_O_4_ particles, and polymerized using azobis­(isobutyronitrile) as the initiator at 60 °C under nitrogen for 24 h. Postpolymerization, the template was removed via Soxhlet extraction using methanol and acetic acid, creating selective recognition sites. A nonimprinted control polymer was synthesized under identical conditions, omitting the template. The resulting Fe_3_O_4_/MIP exhibited a porous, high-surface-area structure with selective binding cavities, which is mixed into a carbon paste.

**Figure 4:**
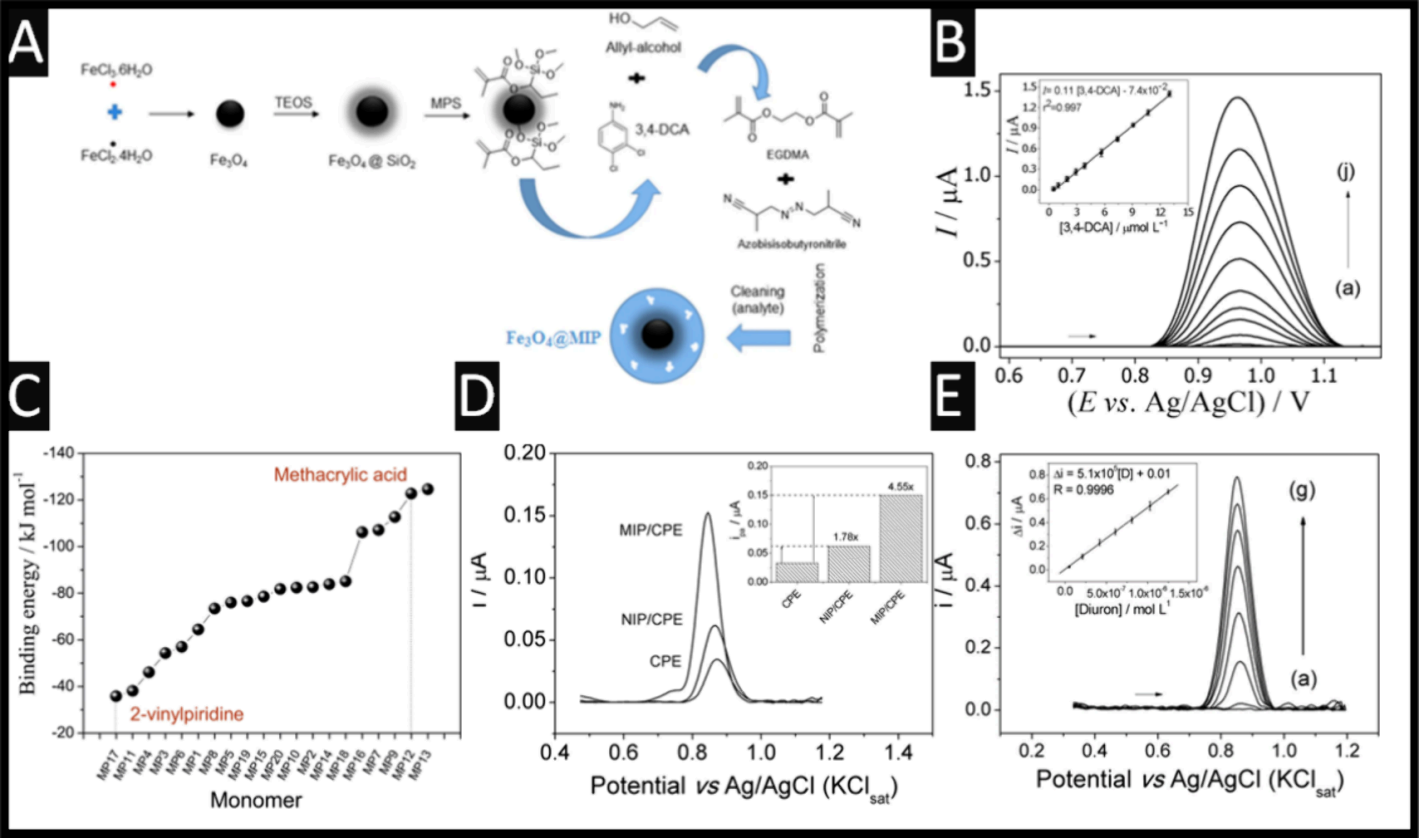
(A) An overview of the formation of magnetic nanoparticles and the associated synthesizes. (B) Square-wave voltammetry of different concentrations of diuron and the analytical curves into a pH of 5. Figure reproduced from reference 54. Copyright 2025 Springer. (C) Study of the monomer–template interaction using computational simulation. M1: bis-acrylamide-N,*N*-methylene; M2: imidazole-4-acrylic acid; M3: ethyl imidazole-4-acrylic acid ester; M4: acrylic acid; M5: acrylamide; M6: acrolein; M7: allylamine; M8: allylamine; M9: ethylene glycol dimethacrylate; M10:2-(cyanoethylamine) ethyl methacrylate; M11: methylene succinic acid; M12: methacrylic acid; M13: methacrylic acid: M14:4-divinylbenzene: M15: styrene: M16:1-vinylimidazole: M17:2-vinylpyridine; M18:4-vinylpyridine: M19:2-acrylamido-2-methyl-1-propane-sulfonic acid: M20:2-hydroxyethyl methacrylate. (D) Square wave voltammetry profiles comparing the response of MWCNT-COOH-MIP/CPE, MWCNT-COOH/CPE, MWCNT/CPE, and CPE toward diuron. Analytical conditions: deposition time: 120 s; potential: +0.2 V (vs Ag/AgCl); pH 8; diuron: 4 × 10^–7^ M. (E) Square-wave voltammetry profiles using the MWCNT-COOH-MIP/CPE composite electrode and associated analytical curve (inset) with error bars (for 3 analytical curves in triplicate). Analytical conditions: deposition time: 120 s; potential: + 0.2 V (vs Ag/AgCl); pH 8; Shown are the concentrations of diuron: (a) 5.2 × 10^–8^, (b) 2.1 × 10^–7^, (c) 4.2 × 10^–7^, (d) 6.1 × 10^–7^, (e) 8.1 × 10^–7^, (f) 1.03 × 10^–6^, and (g) 1.25 × 10^–6^ M. Figure reproduced from reference 18. Copyright 2015 Elsevier.

Figure 4B illustrates the electrochemical response of the Fe_3_O_4_/MIP-modified carbon paste electrode toward increasing concentrations of 3,4-dichloroaniline using square wave voltammetry in pH 5.0. A well-defined anodic peak is observed ∼ +0.92 V (vs Ag/AgCl), corresponding to the irreversible oxidation of 3,4-dichloroaniline. As the analyte concentration increases, the peak current rises proportionally, indicating a clear and stable sensor response. The inset shows the corresponding calibration curve, demonstrating excellent linearity across the range of 0.5 to 16 μM and a LoD of 32 nM. The performance of the sensor was validated through comparison with standard high-performance liquid chromatography with ultraviolet for the detection of spiked 3,4-dichloroaniline in soil, river water, and tap water. The recovery rates obtained using the sensor were close to 100%, with negligible relative errors when compared to the chromatographic results, demonstrating strong agreement between the two techniques. While both methods delivered accurate and reliable quantification, the authors highlight a key practical advantage of the electrochemical approach: significantly reduced analysis time. High-performance liquid chromatography with ultraviolet method requires approximately 1 h for equipment setup and calibration curve generation, whereas the electrochemical method enables rapid analysis and calibration within a matter of minutes. This time efficiency, coupled with low reagent consumption and ease of electrode surface renewal, underscores the suitability of the sensor for fast, on-site monitoring of 3,4-dichloroaniline in environmental matrices.^54^ The use of MIPs should be considered further when one can tailor these to measure both diuron and its derived compound degradation products.

Notable work has been reported by Wong et al.^18^ who have developed a sensor using a MIP and MWCNT-COOH incorporated into a carbon paste electrode, where they have studied the selection of the monomer used in the MIP. Using structural and functional affinity, the interaction of each monomer was explored with the template of diuron using computation simulation. As shown in Figure 4C, one can see the binding energy plotted against monomers where M17­(2-vinylpiridine) has the lowest binding energy, whereas MP 12 (methacrylic acid) had the highest, which was taken forward to be used in the MIP formation toward diuron. Note that the authors did not use MP 13 as this is not a useful polymerization agent.^18^ Using high-performance liquid chromatography and the two monomers transformed into MIPs via bulk polymerization in solution, the authors studied the adsorption performance of diuron where the MIP synthesized with the methacrylic acid monomer showed better adsorption performance, compared to the use of 2-vinylpyridine. This MIP was taken forward to assess its potential as the basis of a sensor toward diuron. The authors studied the different combination of using bare carbon paste electrode, MWCNT/carbon paste electrode, MWCNT-COOH/carbon paste electrode, and NIP (control polymer without the template)/carbon paste electrode, MIP/carbon paste electrode, and MWCNT-COOH/MIP/carbon paste electrode; please see Figure 4D. Clearly, the greatest response is attributed to the MWCNT-COOH/MIP/carbon paste electrode, which can be attributed to enhanced sensor sensitivity, where a 7.9× increase is observed compared to a bare carbon paste electrode surface.^18^ Interestingly, this sensor reports a low LoD of 9 nM and a wide linear range of 0.52 nM–1.25 μM, whereas shown in Figure 4E, one can observe the current rises as the diuron concentration is increased; improved electronic transfer is achieved with the MWCNT-COOH and the MIP achieved enhanced sensitivity.^18^ This sensor is last shown to be useful for the real-sample analysis of spiked diuron in river water samples, which reports recoveries between 96.1 and 99.5%, which is compared with high-performance liquid chromatography. The authors state that the high-performance liquid chromatography can only detect 2.1 × 10^–5^ M, which shows that electrochemical-based approaches provide better sensitivity, lower consumption of reagents, and shorter analysis time; such a sensor should be considered further expanding the samples range to others (e.g., soil, fruits, vegetables, etc.).

## Detection Modalities

Using MIPs with electrochemiluminescence (ECL) has been reported for the sensing of diuron.^33^ ECL is a luminescent phenomenon in which light is emitted from a luminophore as a result of electrochemical reactions occurring on the surface of an electrode. Unlike fluorescence or chemiluminescence, ECL does not require external light sources or chemical oxidants; instead, it relies on the application of a voltage to generate reactive intermediates that form excited-state species. When this species returns to its ground state, it emits light. The process is highly controllable, exhibits low background noise, and offers excellent sensitivity, making it ideal for trace-level detection. ECL has been widely adopted in clinical diagnostics, environmental monitoring, and food safety testing due to its rapid response and ability to operate under mild conditions. Common luminophores used in ECL include ruthenium­(II) complexes, luminol, and nanomaterials such as quantum dots to name just a few.^55–57^ A novel ECL sensor was developed for the sensitive detection of the diuron, integrating gold nanoclusters and MIPs into a dual-quenching detection platform.^33^ The gold nanoclusters were synthesized via a ligand-assisted reduction method at room temperature, using 6-aza-2-thiothymine (ATT) as both the stabilizing and reducing agent. Specifically, an aqueous solution containing ATT (0.08 M) and NaOH (0.2 M) was mixed with an equal volume of the HAuCl_4_ solution (10 mg mL^–^^1^). The reaction was carried out in the dark at room temperature for 1 h, leading to the formation of a yellow solution, indicative of gold nanocluster formation. The resulting product was purified by dialysis for 24 h using a cellulose ester membrane followed by freeze-drying overnight. The dry gold nanoclusters powder was then redispersed in water and stored at 4 °C for subsequent use. This approach produced gold nanoclusters of 2.1 (± 0.3) nm diameter. The use of nanoclusters offers a significantly higher surface-to-volume atom ratio compared to nanoparticles, enhancing their electrochemical reactivity. Moreover, the electronic structure transitions from the bulk-like metallic energy bands characteristic of nanoparticles to discrete molecular orbital levels in nanoclusters, resulting in unique redox behavior and quantum-size effects. These ATT-protected gold nanoclusters are drop-cast onto a glassy carbon electrode. Next, the MIP was fabricated directly on the gold nanocluster-modified glassy carbon electrode through an in situ electropolymerization process. After drop-casting, the electrode was immersed in buffer solution of pH 7.4 containing thiophene as the functional monomer and diuron as the template molecule. Electropolymerization was carried out by cycling the potential between 0 and +1.8 V (vs Ag/AgCl) for 15 cycles, leading to the formation of a polymer film on the electrode surface with diuron embedded in its matrix. Following polymerization, the electrode was washed in an ethanol/water solution (9:1, v/v) for 35 min to effectively remove diuron and reveal specific recognition cavities complementary in shape and functionality to the template. Using triethylamine (TEA), as the coreactant that facilitates light emission in the presence of gold nanoclusters (AuNCs), one can observe the response of the sensor to increasing concentrations added to a pH 7.4 buffer. The response decreases and the corresponding calibration curves; see Figure 5A­(i) and (ii), noting that the author used Δ*E*_*ECL*_ = *I*_0_ – *I* where *I*_0_ is the ECL intensity of the sensor after *elution* of diuron (i.e., when the imprinted cavities are empty and the signal is at its maximum) and *I* is the ECL intensity after *rebinding* diuron to the MIP cavities i.e., when diuron quenches the ECL signal.

**Figure 5:**
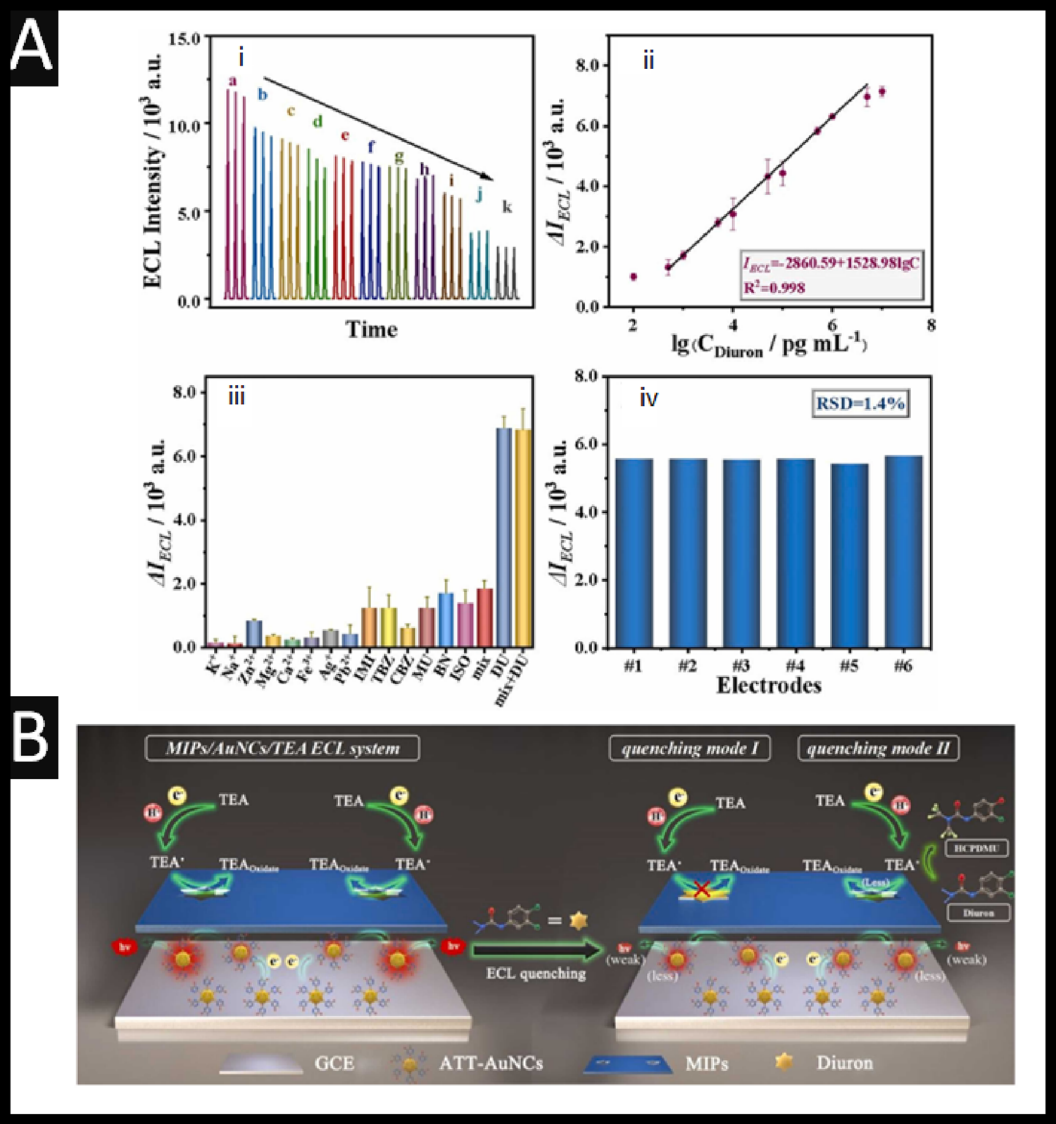
(A) (i) ECL responses of the MIP-ECL sensor with different concentrations of diuron from 1.00 × 10^–10^ to 1.00 × 10^–5^ g mL^–1^. (ii) Calibration curve of the ECL responses with respect to diuron concentration. (iii) Selectivity and (iv) reproducibility for the proposed MIP-ECL sensor for diuron detection. (B) Dual-quenching mechanisms of the fabricated MIP-ECL sensor by diuron: (mode I) “blocking effect”, and (mode II) interaction between diuron and TEA. Figure reproduced from.^33^ Copyright 2024 Elsevier.

The sensor showed excellent analytical performance with a detection limit of 2.16 × 10^–12^ g mL^–^^1^ and wide linear range. A range of potential interferents were evaluated to assess the selectivity of the sensor toward diuron; see Figure 5Aiii, which shows that the tested interferents include common metal ions (K^+^, Na^+^, Mg^2^^+^, Ca^2^^+^, Fe^3^^+^, Pb^2^^+^, Zn^2^^+^) and organic compounds (benzene hexachloride, thiabendazole, carbendazim, metolachlor, bifenthrin), where each interferent was tested at a concentration 10 times higher than that of diuron. This shows that the ECL signal for diuron is significantly higher than for any of the individual interferents or the mixture containing all of them, indicating that the sensor has high selectivity for diuron and that the presence of these potentially coexisting substances does not significantly interfere with the detection signal.^33^

The dual-quenching strategy employed in the MIP-ECL sensor for diuron detection operates through two synergistic mechanisms, each contributing to suppression of the ECL signal. The first mechanism, known as the “blocking effect,” occurs when diuron binds to its specific recognition sites within the MIP layer; see Figure 5B. This binding physically hinders the access of the coreactant, triethylamine (TEA), to the electrode surface, thereby impeding the electron transfer between electrochemically oxidized gold nanoclusters (AuNCs•^+^) and TEA radicals (TEA•). The electron transfer process that normally generates excited-state species AuNCs* is thus reduced. This can be represented by the reactions:

$$\mathrm{AuNCs}-e^{-}\to {\mathrm{AuNCs}}^{•+}$$ 1

$$\mathrm{TEA}-e^{-}\to {\mathrm{TEA}}^{•+}\to {\mathrm{TEA}}^{•}+H^{+}$$ 2

$${\mathrm{AuNCs}}^{•+}+{\mathrm{TEA}}^{•}\left(\mathrm{less}\right)\to {\mathrm{AuNCs}}^{*}\left(\mathrm{less}\right)+{\mathrm{TEA}}_{\mathrm{Oxidate}}$$ 3

$${\mathrm{AuNCs}}^{*}\left(\mathrm{less}\right)\to \mathrm{AuNCs}+hv\left(\mathrm{weak}\right)$$ 4

In the presence of diuron, the amount of TEA• available for reaction (Equation 3) is diminished by both physical blocking and chemical consumption. The second quenching mechanism (see Figure 5B) involves a direct interaction between diuron and TEA•, in which diuron undergoes a hydroxylation reaction catalyzed by electrochemical stimulation, forming a hydroxylated product, HCPDMU (3-hydroxychlorophenyl-dimethylurea):

$$C_{9}H_{10}C_{l2}N_{2}O\left(\mathrm{diuron}\right)+{\mathrm{TEA}}^{•}\to \mathrm{HCPDMU}+{\mathrm{TEA}}_{\mathrm{Oxidate}}$$ 5

This reaction not only consumes TEA•, reducing its availability for ECL generation, but also leads to secondary quenching through further oxidation of HCPDMU to a benzoquinone-like species capable of accepting electrons from TEA•. As a result, both the formation of AuNCs* and the intensity of emitted light (hν, Equation 4) are significantly reduced. Together, these two pathways define the dual-quenching mechanism, enabling highly sensitive detection of diuron via a concentration-dependent decrease in ECL signal.^33^ To evaluate the practical applicability of the developed MIP-ECL sensor, water samples were collected from the River Navigation Grand Canal and analyzed using the standard addition method. The sensor successfully detected diuron at concentrations as low as 400 pg·mL^–^^1^, a level at which conventional high-performance liquid chromatography coupled with mass spectrometry failed to produce a detectable signal, highlighting the superior sensitivity of the ECL-based method. Recovery rates for the spiked samples ranged from 94.6% to 103%, with relative standard deviations between 3.4% and 5.8%, demonstrating both high accuracy and reproducibility. This ECL based sensor has the potential for ultrasensitive, rapid, and portable electrochemical sensing of diuron.

## Conclusions

We have summarized the electrochemical platforms reported for the measurement of diuron, highlighting how electrochemical methods offer a compelling alternative to traditional laboratory-based techniques. While laboratory instruments provide high precision and are well-suited for confirmatory analysis, they often require lengthy sample preparation, complex operation, and extended analysis times—typically close to an hour per sample. In contrast, electrochemical sensors enable rapid detection and calibration within minutes with minimal sample preparation and lower operational costs. As stated above, where for example, diuron is not fully banned but is classified as a restricted-use pesticide, we believe that useful electrochemical based sensors can measure across the range of 10 nM–10 μM with LODs lower than 10 nM. From inspection of Table 1, linear ranges are in the order of nM−μM where low LoD are at nM levels, and for example, the lowest linear range and LoD is attributed to the use of ECL.^33^ This sensor has been validated against high-performance liquid chromatography coupled with mass spectrometry, which shows merit for this to achieve in-field sensing. Electrochemical sensors demonstrate selectivity even in complex matrices, making them particularly well-suited for on-site, real-time environmental monitoring of diuron. These advantages position electrochemical sensing as a valuable complement to traditional methods, offering practical solutions for field applications.

However, there remain several limitations to address:

Simultaneous detection: only a few electroanalytical sensors have reported the simultaneous detection of diuron alongside related herbicides. This remains a challenge that should be addressed, for example, the development of MIPs tailored for multiplex detection are a possibility.
Degradation products: the electrochemical detection of diuron’s degradation products is largely unexplored. Given their potential toxicity, for example, note that 3,4-dichloroaniline has a higher toxicity than diuron,^2^ this area is critical and warrants further investigation.
Sample pretreatment strategies: as shown in Table 1, a range of pretreatment approaches have been employed depending on the sample type. For environmental samples (e.g., river, sea, tap waters), simple approaches have been used where they filter, dilute, and spike with diuron. For more complex matrices such as soil and fruits and vegetables, solid–liquid extraction approaches are typically used prior to spiking with diuron. As the field advances and the range of samples broadens, more complex, characterized by high organic matter, diverse chemical speciation, and the presence of interfering ions, there is the need to utilize additional pretreatment steps. For example, core–shell magnetic MIPs have been shown to selectively bind to diuron for example in the complex matrices of paddy field water and paddy soil.^58^ Other useful approaches have utilized on-chip microfluidic systems that integrate sample pretreatment steps such as filtration, extraction, or selective binding offering a compact and automated approach for handling complex matrices with minimal manual intervention; both should be considered further.
Real-world samples: as shown in Table 1, sample matrices have included water, vegetables, fruits, juices, and soil. However, the majority of studies rely on spiked samples rather than naturally contaminated ones. Researchers must focus on real-world sample analysis and compare results with established laboratory-based techniques to validate sensor performance.
Field-deployable technologies: as can be seen from inspection of Table 1, the use of screen-printed sensors are limited. Researchers should continue to enhance the field by the further development of screen-printed and associated fabrication approaches (e.g., thick film, inkjet, 3D printing etc.) to enable the realization of in-field sensors compatible with smartphone-based readers and ultralow-cost potentiostats.

Finally, most electrochemical sensors for diuron have yet to be evaluated under true in situ field conditions, where environmental variables such as temperature, humidity, and complex matrices may significantly impact sensor performance. Addressing these challenges is essential for developing robust, field-deployable electroanalytical sensors capable of reliable environmental monitoring for the measurement of diuron.
